# Frontal and superior temporal auditory processing abnormalities in schizophrenia^☆^

**DOI:** 10.1016/j.nicl.2013.05.002

**Published:** 2013-05-15

**Authors:** Yu-Han Chen, J. Christopher Edgar, Mingxiong Huang, Michael A. Hunter, Emerson Epstein, Breannan Howell, Brett Y. Lu, Juan Bustillo, Gregory A. Miller, José M. Cañive

**Affiliations:** a University of New Mexico School of Medicine, Department of Psychiatry, Albuquerque, NM, USA; b New Mexico Raymond G. Murphy VA Healthcare System, Psychiatry Research, Albuquerque, NM, USA; c Children's Hospital of Philadelphia and University of Pennsylvania, Philadelphia, PA, USA; d University of California San Diego, Department of Radiology, San Diego, CA, USA; e San Diego VA Healthcare System, Department of Radiology, San Diego, CA, USA; f University of New Mexico, Department of Psychology, Albuquerque, NM, USA; g University of Hawaii at Manoa, Department of Psychiatry, Honolulu, HI, USA; h University of Delaware, Department of Psychology, USA

**Keywords:** DTI, diffusion tensor imaging, ECG, electrocardiogram, EEG, electroencephalography, EOG, electro-oculogram, ERP, event-related potential, ERF, event-related field, fMRI, functional magnetic resonance imaging, FDR, false discovery rates, HC, healthy controls, IFG, inferior frontal gyrus, ITG, inferior temporal gyrus, MEG, magnetoencephalography, PANSS, Positive and Negative Syndrome Scale, PFC, prefrontal cortex, S1, first click, S2, second click, SES, socioeconomic status, SFG, superior frontal gyrus, SMA, supplementary motor area, SMG, supramarginal gyrus, sMRI, structural magnetic resonance imaging, SSS, Signal Space Separation, STG, superior temporal gyrus, VESTAL, Vector-based Spatio-temporal Analysis using L1-minimum norm, Schizophrenia, Auditory, Superior temporal gyrus, Frontal cortex, MEG

## Abstract

**Background:**

Although magnetoencephalography (MEG) studies show superior temporal gyrus (STG) auditory processing abnormalities in schizophrenia at 50 and 100 ms, EEG and corticography studies suggest involvement of additional brain areas (e.g., frontal areas) during this interval. Study goals were to identify 30 to 130 ms auditory encoding processes in schizophrenia (SZ) and healthy controls (HC) and group differences throughout the cortex.

**Methods:**

The standard paired-click task was administered to 19 SZ and 21 HC subjects during MEG recording. Vector-based Spatial–temporal Analysis using L1-minimum-norm (VESTAL) provided 4D maps of activity from 30 to 130 ms. Within-group t-tests compared post-stimulus 50 ms and 100 ms activity to baseline. Between-group t-tests examined 50 and 100 ms group differences.

**Results:**

Bilateral 50 and 100 ms STG activity was observed in both groups. HC had stronger bilateral 50 and 100 ms STG activity than SZ. In addition to the STG group difference, non-STG activity was also observed in both groups. For example, whereas HC had stronger left and right inferior frontal gyrus activity than SZ, SZ had stronger right superior frontal gyrus and left supramarginal gyrus activity than HC.

**Conclusions:**

Less STG activity was observed in SZ than HC, indicating encoding problems in SZ. Yet auditory encoding abnormalities are not specific to STG, as group differences were observed in frontal and SMG areas. Thus, present findings indicate that individuals with SZ show abnormalities in multiple nodes of a concurrently activated auditory network.

## Highlights

• Auditory encoding in schizophrenia (SZ) and healthy controls (HC) was examined.
• Distributed source localization provided whole-brain measures from 30 to 130 ms.
• Abnormalities were observed in superior temporal gyrus (STG) auditory areas in SZ.
• Encoding abnormalities were also observed in frontal and supramarginal gyrus areas.
• SZ shows abnormalities in multiple nodes of a distributed auditory network.

## 1 Introduction

Using electroencephalography (EEG) and magnetoencephalography (MEG), a now large number of studies show smaller 100 ms auditory amplitudes in individuals with schizophrenia (SZ) than healthy controls (HC). In a review of studies examining N1 and M100 in schizophrenia, Rosburg et al. (2008) concluded that 100 ms auditory abnormalities are most commonly observed in studies using interstimulus intervals greater than 1 s and that an increase in N1 amplitude by allocation of attention is often lacking in individuals with SZ. Several large-sample studies provide examples. Examining N1 activity in the standard paired-click paradigm, Turetsky et al. (2008) observed a small first and a normal second N1 click response in SZ (N = 142) relative to HC (N = 221). Reduced N1 was also observed in the unaffected first-degree relatives of individuals with SZ without co-morbid psychiatric or substance use conditions, and N1 amplitude was observed to be a heritable measure and a better endophenotype than N1 gating. In another recent large-N study, Smith et al. (2010) used simultaneous EEG and MEG to examine 100 ms auditory processes in individuals with SZ (N = 79) and HC (N = 73) during a paired-click task. Patients had larger N1 Cz and left and right superior temporal gyrus (STG) M100 ratio scores (second-click/first-click), with EEG and MEG ratio score group differences due to a smaller first click (S1) response in patients, suggesting a deficit in encoding auditory information rather than a deficit in filtering redundant information.

N1 (EEG) and M100 (MEG) are the most prominent deflections of the adult auditory event-related potential (ERP) or field (ERF) (Hari, 1990). In an early study, Naatanen and Picton (1987) argued that the electric N1 reflects contributions from up to 6 distinct cortical areas: dipoles in or near the primary auditory cortex as well as prefrontal cortex (PFC) sources. Later studies showed connections between STG and PFC. For example, several studies have demonstrated bidirectional connections between STG and PFC in the rhesus monkey (Knight et al., 1999). Combined tracing and immunohistochemistry studies have revealed that projections from PFC pyramidal neurons make synaptic contact with a subset of calbindin-positive GABAergic interneurons in auditory areas (Barbas et al., 2005). Through these connections, PFC pyramidal neurons may modulate the excitability of microcircuits within the monkey's auditory belt and parabelt (Barbas et al., 2005). Neural tracers infused into auditory cortex have also been found to emerge in PFC axonal terminal (Romanski, 2004; Romanski et al., 1999). Finally, anatomical studies in humans have identified white-matter tracks connecting auditory cortex with lateral and medial PFC. These observations have been corroborated via in vivo imaging (Catani et al., 2002). Taken together, monkey and human studies support the hypothesis that PFC pyramidal neurons modulate the flow of information in auditory cortices by controlling the activity of GABAergic interneurons, which in turn modulate the excitability of STG pyramidal neurons (Barbas et al., 2005). With regard to individuals with SZ, there is evidence of aberrant fronto-temporal connectivity: in a diffusion tensor imaging (DTI) study, Abdul-Rahman et al. (2012) showed that disruption of fronto-temporal white-matter tracks involving arcuate fasciculus may be associated with psychotic features and auditory hallucinations in SZ.

Although equivalent current dipole source localization techniques work well to examine 50 and 100 ms STG activity (Edgar et al., 2003, 2008; Huang et al., 2003; Smith et al., 2010), equivalent current dipole techniques are likely less optimal in terms of localizing auditory activity in non-STG areas, because activity in non-STG areas is often distributed and thus non-dipolar. The present study reports findings using a lead-field-based source localization method, Vector-based Spatio-temporal Analysis using L1-minimum norm (VESTAL; Huang et al., 2006), to examine auditory processes throughout the brain in HC and in individuals with SZ. Given that our and others previous paired-click findings indicated group differences for the S1 but not the second click (S2)^1^ (Smith et al., 2010; Turetsky et al., 2008), the present study focused on examining early S1 activity at 50 ms and 100 ms. The following predictions were made:

Hypothesis 1 STG activity would be observed in both groups, and VESTAL STG group differences would be analogous to those reported previous studies. In particular, 100 ms STG group differences would be observed bilaterally. If 50 ms group S1 differences were observed, the 50 ms group differences would be left lateralized.

Hypothesis 2 Given studies indicating prefrontal activation during simple auditory tasks, frontal activation was expected in both groups. Although prior literature does not provide evidence for making strong predictions about group differences in frontal activity, it was hypothesized that the spatial pattern of frontal activity would be different in patients and controls.

Although equivalent current dipole source localization techniques work well to examine 50 and 100 ms STG activity (Edgar et al., 2003, 2008; Huang et al., 2003; Smith et al., 2010), equivalent current dipole techniques are likely less optimal in terms of localizing auditory activity in non-STG areas, because activity in non-STG areas is often and thus non-dipolar. The present study reports findings using a lead-field-based source localization method, Vector-based Spatio-temporal Analysis using L1-minimum norm (VESTAL; Huang et al., 2006), to examine auditory processes throughout the brain in HC and in individuals with SZ. Given that our and others previous paired-click findings indicated group differences for the S1 but not the second click (S2)^1^ (Smith et al., 2010; Turetsky et al., 2008), the present study focused on examining early S1 activity at 50 ms and 100 ms. The following predictions were made:

Hypothesis 1 STG activity would be observed in both groups, and VESTAL STG group differences would be analogous to those reported previous studies. In particular, 100 ms STG group differences would be observed bilaterally. If 50 ms group S1 differences were observed, the 50 ms group differences would be left lateralized.

Hypothesis 2 Given studies indicating prefrontal activation during simple auditory tasks, frontal activation was expected in both groups. Although prior literature does not provide evidence for making strong predictions about group differences in frontal activity, it was hypothesized that the spatial pattern of frontal activity would be different in patients and controls.

## 2 Methods and materials

### 2.1 Subjects

Nineteen patients with chronic SZ (14 males, mean age 40.31 ± 11.7 years) and 22 age-matched HC (15 males; mean age 34.95 ± 10.2 years) were recruited. Selection criteria were (1) diagnosis of schizophrenia with no other Axis I diagnosis, determined by the Structured Clinical Interview for DSM-IV-Patient Edition (SCID-DSM-IV; American Psychiatric Association, 1994); (2) stable, continuous treatment with one antipsychotic medication for at least 3 months; (3) no history of substance dependence (determined during the SCID-DSM-IV interview); (4) no history of alcohol or other substance abuse in the past 3 months (determined during the SCID-DSM-IV interview); (5) no history of head injury with loss of consciousness for more than 5 minutes; and (6) no psychiatric hospitalization in the last 3 months. As shown in Table 1, groups did not differ in age, education, or parental socioeconomic status (SES, Oakes and Rossi, 2003; scores derived from individual's income, education, and occupation information, with lower SES score indicating higher socioeconomic status). Patients' SES was significantly lower than controls'. Mean total scores of the Positive and Negative Syndrome Scale (PANSS) (Kay et al., 1987) were 20.00 for positive symptoms and 17.46 for negative symptoms (N = 13; PANSS scores were not available in 6 subjects). Additional recruitment procedures and additional information on inclusion and exclusion criteria are reported in Smith et al. (2010).

Five HC and 2 SZ were left-handed as assessed by the Waterloo Handedness Questionnaire (Bryden, 1977). Patients with SZ were medicated and clinically stable without change in medications for at least one month before MEG. In the patient group, 14 participants were treated with 2nd generation antipsychotics: 2 on aripiprazole, 5 on olanzapine, 3 on risperidone, 3 on quetiapine and 1 on ziprasidone. Two participants were treated with 1st generation antipsychotic halperiodol. Finally, 2 subjects were treated with both aripiprazole and clozapine and 1 with aripiprazole, clozapine, and halperiodol. The average of Chlorpromazine equivalent dosage for all patients was 587 mg/day (1 patient did not have medication dosage information). Six patients with SZ and 2 HC were smokers.

### 2.2 Paired-click paradigm

The paired-click paradigm followed the protocol of Adler et al. (1993), in which 3 ms binaural clicks were presented in pairs (S1 and S2) with 500 ms inter-stimulus interval and with inter-trial interval jitter between 7 and 11 s, averaging 9 s. Clicks were delivered through earphones placed in each ear canal. The peak intensity of the click was presented 35 dB above each subject's hearing threshold. Presenting 150 click trials, the duration of the task was approximately 25 minutes. As previously noted, the present study examined only S1 activity at 50 and 100 ms.

### 2.3 MEG and MRI data acquisition and coregistration

MEG data were recorded in a magnetically shielded room (Vacuumschmelze, Germany) using a 306-channel Vector-View MEG system (Elekta-Neuromag, Helsinki, Finland). After a band-pass (0.1–330 Hz) and 60 Hz notch filter, MEG signals were digitized at 1000 Hz. Electro-oculogram (EOG) (vertical EOG on the upper and lower left sides) and electrocardiogram (ECG) (at the collarbone) were also obtained. The subjects' head position was monitored using four HPI coils attached to the scalp. Participants were asked to refrain from smoking for at least 1 h before the recording session. To ensure compliance, they were asked to report to the facility an hour before recording commenced, during which time participants were familiarized with equipment and procedures. After the MEG session, structural magnetic resonance imaging (sMRI) provided T1-weighted, 3-D anatomical images using a 3 T Siemens Trio scanner (voxel size 1 × 1 × 1 mm^3^).

To coregister MEG and sMRI data, three anatomical landmarks (nasion and right and left preauriculars) as well as an additional 150 + points on the scalp and face were digitized for each subject using the Probe Position Identification (PPI) System (Polhemus, Colchester, VT). The three fiducials were identified in the subject's sMRI, and a transformation matrix that involved rotation and translation between the MEG and sMRI coordinate systems was obtained by matching the 150 + points from the PPI measurements to the surfaces of the scalp and face from the sMRI.

### 2.4 Magnetic source analysis

MEG raw signals were first processed with Signal Space Separation (SSS; Taulu et al., 2004) using Maxfilter (Elekta MaxfilterTM; Elekta Oy). SSS separates neuronal magnetic signals arising from inside the MEG sensor array from external magnetic signals arising from the surrounding environment to effectively reduce environmental noise and artifacts. After SSS, S1 epochs 500 ms pre-stimulus to 500 ms post-stimulus were averaged. Trials containing eye-blinks and large eye-movements were excluded. On average, 103 trials were obtained for each subject, and there were no group differences in number of accepted trials (t(39) = 0.30, *p* = 0.77).

To calculate MEG forward solutions, a realistically shaped Boundary Element Method (BEM) head model was created from each subject's inner skull (Hamalainen and Sarvas, 1989), with the BEM mesh obtained from tessellating the inner skull surface from the MRI into ~ 6000 triangular elements with ~ 5 mm size. Vector-based Spatio-temporal Analysis using L1-minimum norm (VESTAL; Huang et al., 2006) provided source images for each subject. VESTAL selects the source configuration that minimizes the absolute value of the source strength. Both magnetometers and planar gradiometers were used in the source localization. Whereas L1-minimum norm methods have been used in previous MEG studies (Auranen et al., 2005; Osipova et al., 2005; Pulvermuller and Shtyrov, 2003), a major limitation with previous L1-minimum norm routines has been instability in spatial localization and poor smoothness in reconstructed source time-courses (i.e., the time-course of one specific grid point can show substantial spiky-looking discontinuities). This problem is also encountered in other focal localization methods using lead-field approaches. In VESTAL, the temporal information in the data is used to enhance the stability of the reconstructed solution. Since this approach makes no assumptions about the temporal dynamics of the sources, the approach can handle sources that are 100% correlated. VESTAL also effectively obtains source strength and dipole orientation without iteration or choosing a pre-fixed dipole orientation for each grid node. The technical details of VESTAL, in which VESTAL was tested with computer simulations and human data, are presented in Appendix A and in Huang et al. (2006). Results show that VESTAL provides high spatial stability and continuous temporal dynamics, without compromising spatial or temporal resolution.

**Fig. 1:**
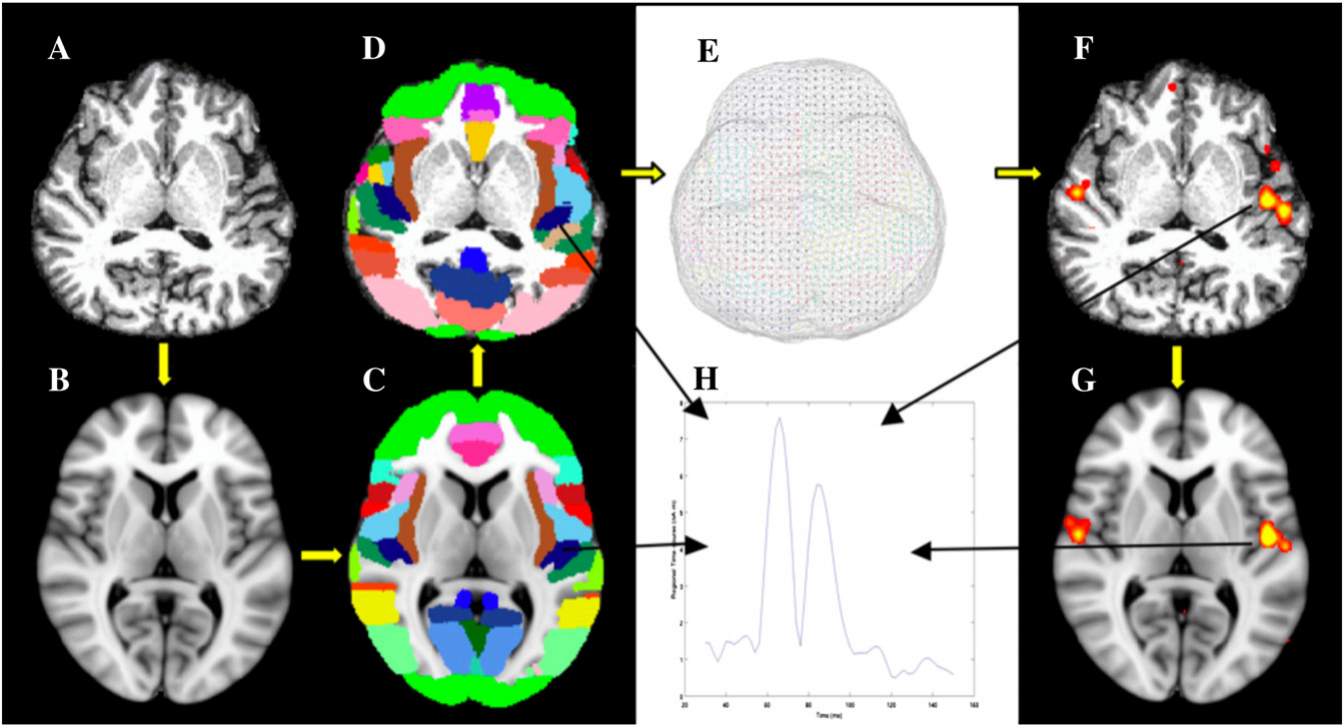
VESTAL data processing stream. (A) T1-MRI from an individual subject; (B) MNI-152 Atlas space; (C) cortical mask from MNI-152; (D) cortical mask transferred back to the individual MRI space; (E) VESTAL source grid with cortical and subcortical regions. Gray triangles are BEM mesh for MEG forward calculation; (F) VESTAL source image of the subject's auditory response overlayed on the T1-MRI; (G) VESTAL activity transferred to the MNI-152 coordinates; (H) regional time-course from VESTAL results.

For group analyses, the following procedures were applied. (1) T1-weighted sMRIs from each subject (Fig. 1A) were registered to MNI space (Montreal Neurological Institute, MNI-152 atlas as in Fig. 1B) using an affine transformation (FLIRT–FMRIB's Linear Image Registration Tool) (Jenkinson and Smith, 2001) in FSL (www.fmrib.ox.ac.uk/fsl/). (2) The cortical (Fig. 1C) and subcortical masks with pre-defined brain regions from the standard atlas were transferred to the individual's headspace (Fig. 1D), using the inverse of the transformation obtained in the first step: the Harvard-Oxford Atlas, part of the FSL software with masks of 96 cortical gray-matter regions (48 regions in each hemisphere), 21 sub-cortical regions, and cerebellum, was used. (3) The regional masks were down-sampled to a cubic source grid with voxels of 5 mm per side (Fig. 1E). (4) VESTAL MEG source imaging used the source grid from step 3. This step permits group-based analyses. In the shown example, MEG responses evoked by S1 localized to left and right Heschl's gyri (Fig. 1F). (5) Finally, for regions of interest (ROIs), the source time course was obtained by summing activity from all ROI voxels. Fig. 1H shows the time course from left Heschl's gyrus (dark blue region in Fig. 1C and D).

Prior to VESTAL analyses, a 5–55 Hz bandpass filter was applied. VESTAL analyses examined activity 30–130 ms post-stimulus producing a 4D activation map (3D volumes across time) as well as a 2D source time-course matrix. The average percent variance explained for gradiometer data using VESTAL was 95.81% for HC and 94.38% for SZ. The average percent variance explained for magnetometer data using VESTAL program was 96.24% for HC and 93.17% for SZ. There were no group differences in percent variance explained for gradiometer data (t(39) = 1.16, *p* = 0.25) or magnetometer data (t(39) = 1.31, *p* = 0.20).

### 2.5 Statistics

The present analysis examined 50 to 100 ms S1 activity only in cortical regions using source strength from VESTAL volumes summed from 30 to 80 ms and from 80 to 130 ms. Within-group t-tests compared 50 and 100 ms activity to baseline, and between-group t-tests examined group differences. For within-group analyses, spatial smoothing of sigma 2 mm was applied. Given that between group differences are likely smaller than within-group differences for pre- versus post-stimulus activity, spatial smoothing of sigma 5 mm was applied for between-group analyses. To control for multiple comparisons, false discovery rates (FDR; Benjamini, 2010; Benjamini and Hochberg, 1995) were computed for within- and between-group VESTAL statistical images. An FDR of < 1% was set for within-group analysis (FDR q < 0.01), and an FDR of < 5% was set for between-group analysis (FDR q < 0.05), and only voxels that survived a threshold according to q < 0.01 or q < 0.05 were retained for statistical inferences.

## 3 Results

### 3.1 Within-group analyses

**Fig. 2:**
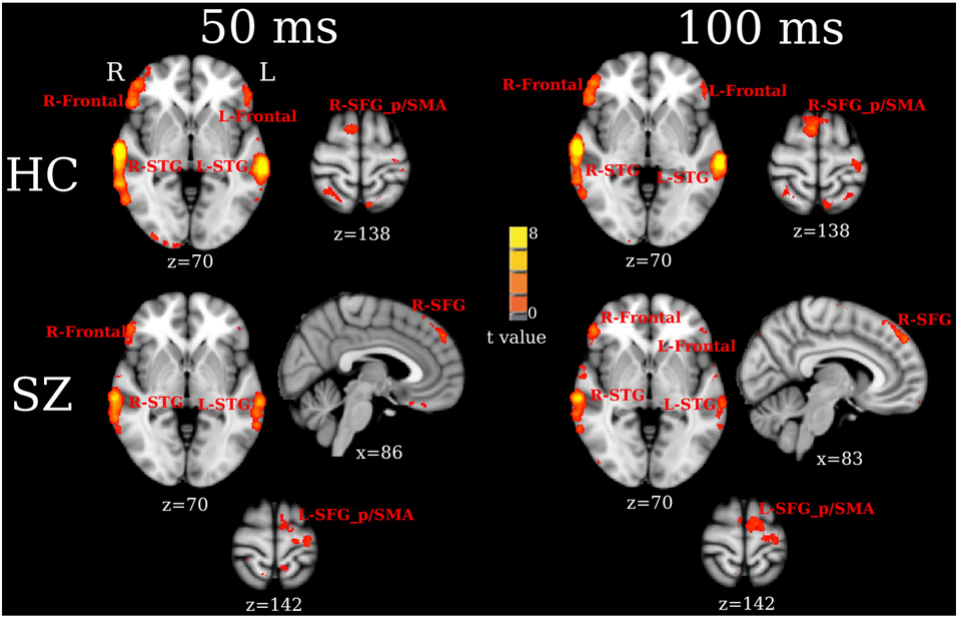
Within-group VESTAL statistics for 50 (left panel) and 100 ms (right panel) activity showing significantly activated areas for HC (top panel) and SZ (bottom panel) (thresholded at FDR q < 0.01).

Fig. 2 shows pre- to post-stimulus 50 and 100 ms maps for each group (FDR q < 0.01). STG and frontal activity was observed bilaterally in the HC and SZ groups at both 50 and 100 ms. In addition, activity in posterior superior frontal gyrus (SFG_p)/supplementary motor area (SMA) was observed in the right hemisphere for HC and in the left hemisphere for SZ. Interestingly, right superior frontal gyrus (R-SFG) activity was observed only in SZ.

### 3.2 Between-group analyses

**Fig. 3:**
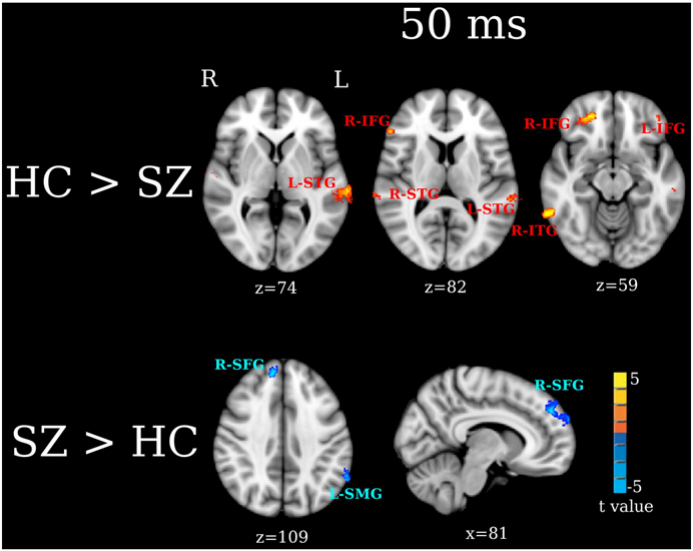
Between-group analyses for 50 ms activity. Activation clusters in yellow/red (thresholded at FDR q < 0.05) show stronger activity in HC than SZ (HC > SZ). Activation clusters (thresholded at FDR q < 0.05) in blue show stronger activity in SZ than HC (SZ > HC). The effect sizes for L-STG, R-Frontal and R-SFG M50 measures are provided in Table 2.

Group contrast maps at 50 ms (Fig. 3) showed group differences in STG and non-STG regions (FDR q < 0.05). HC had stronger left and right STG (L-STG, R-STG), right inferior temporal gyrus (R-ITG), and left and right IFG (L-IFG, R-IFG) 50-ms activity than SZ. SZ had stronger right superior frontal gyrus (R-SFG) and left supramarginal gyrus (L-SMG) 50 ms activity than HC.

**Fig. 4:**
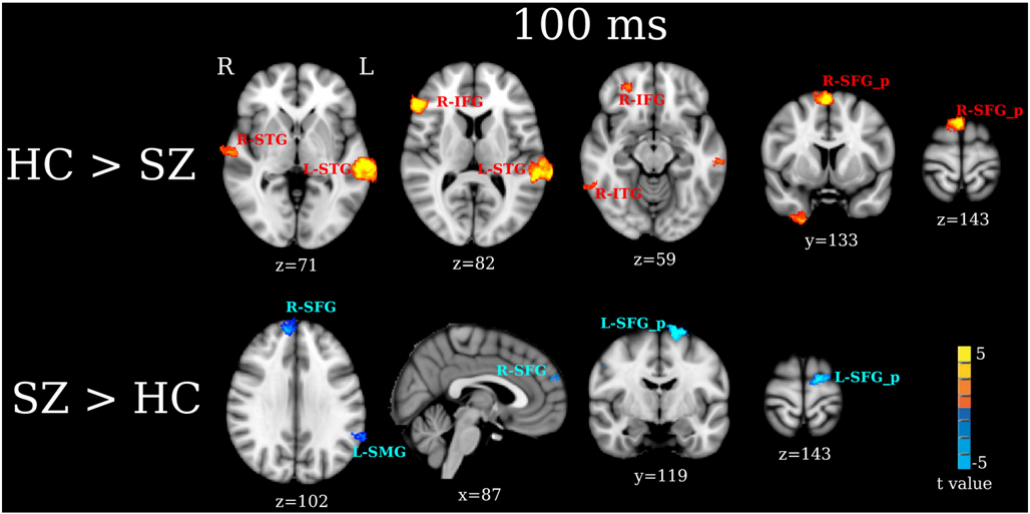
Between-group analyses for 100 ms activity. Activation clusters in yellow/red show stronger activity in HC than SZ (HC > SZ). Activation clusters in blue show stronger activity in SZ than HC (SZ > HC). The effect sizes for L-STG, R-Frontal and R-SFG M100 measures are provided in Table 2.

Similar to the 50 ms group maps, group contrast maps at 100 ms (Fig. 4) showed group differences in STG and non-STG regions (FDR q < 0.05). HC had stronger activity in L-STG, R-STG, R-ITG, R-IFG, and the posterior part of right SFG (R-SFG_p)/SMA than SZ at 100 ms. SZ had stronger activity in R-SFG, posterior part of left SFG (L-SFG_p)/SMA, and L-SMG than HC at 100 ms.

## 4 Discussion

The present study shows that STG and non-STG areas (e.g., frontal regions) are involved in early auditory encoding. Replicating findings from our prior study that used single dipole source localization to examine STG activity (Smith et al., 2010) and now examining a new sample using a different MEG system and applying distributed source localization methods, reduced S1 STG activity was observed in SZ, again supporting impaired encoding of auditory information. Whereas in Smith et al. we observed left 50 ms and bilateral 100 ms STG group differences, group differences were observed bilaterally at both 50 and 100 ms in this new sample.

Present STG findings are consistent with theories postulating basic sensory processing abnormalities as central to schizophrenia (for a review, see Javitt, 2009). As also detailed in Javitt (2009), other studies indicate a downstream consequence of early auditory abnormalities, such that basic auditory abnormalities in schizophrenia are associated with impaired performance on tests of attention (Smith et al., 2010) and tests of prosody (Leitman et al., 2005, 2006). Finally, studies observing that 100 ms responses are associated with decreased STG gray matter (Edgar et al., 2012), as well as N-methyl-d-asparate (NMDA) dysfunction (Javitt et al., 2000), suggest a biological mechanism for encoding abnormalities in schizophrenia.

As noted in the Introduction, Naatanen and Picton (1987) argued that the electric N1 reflects contributions from frontal sources as well as from primary auditory cortex. Present results provide confirmation of these findings, with frontal activity observed in both groups (Fig. 2). Findings are also consistent with imaging studies that have observed frontal activity during auditory tasks.^2^ For example, examining auditory responses in patients with epilepsy using intracranial microelectrode grids, Korzyukov et al. (2007) detected 50 ms temporal and frontal auditory activity, findings consistent with earlier corticography studies suggesting frontal contributions to P50 (Grunwald et al., 2003). Boutros et al. (2011) also detected 100 ms temporal and frontal auditory activity using subdural electrodes. Similar to present findings in controls, Boutros et al. observed activity in the posterior part of STG and in left ventral prefrontal cortex (more exact comparisons between the two studies in terms of frontal activity are difficult, as many subjects in Boutros et al. did not have electrodes placed in anterior frontal regions). Boutros et al. (2011) also observed 100 ms auditory activity in middle temporal gyrus, parietal, cingulate, and occipital regions. Activity observed in similar but not identical locations in these other areas in the present study may be due to the significant latency variability Boutros et al. (2011) observed in several regions. Variability across subjects in the location of activation may also account for study differences.

In the present study, two abnormalities in frontal activity were observed in SZ. First, stronger right 50 and 100 ms IFG activity was observed in HC than SZ. Second, stronger right SFG 50 and 100 ms activity was observed in SZ than HC (Figs. 3 and 4). This pattern of decreased inferior frontal activity but increased superior frontal activity in SZ suggests abnormal activation of fronto-temporal auditory networks in SZ. Recent studies have shown functionally distinct auditory pathways in humans in particular distinct ‘what/where’ auditory system pathways analogous to the ‘what/where’ visual system pathways. For example, Romanski et al. (1999), combining microelectrode recordings with neural tracers infused in auditory cortex of rhesus monkeys, found paths from posterior and anterior auditory cortex that differentially targeted non-spatial (ventral) and spatial (dorsal) frontal areas. Romanski et al. (1999) hypothesized these to be analogous to the ‘what’ and ‘where’ visual pathways. These non-human primate findings are consistent with language processing models, with a ventral stream mapping acoustic speech to conceptual and semantic representations and a dorsal stream mapping phonological information within the frontal articulatory system (Hickok and Poeppel, 2007; McClelland and Rogers, 2003).

Present results suggest that HC activate the ventral ‘what’ auditory pathway more strongly than SZ. Ventral PFC (orbitofrontal cortex) recordings indicate that cells in this region are responsive to the features of complex sounds (Romanski, 2004). In addition, combining fMRI and DTI to identify anatomical pathways associated with language, Saur et al. (2008) found that linguistic processing of sound to meaning requires interaction between temporal lobe and ventrolateral PFC via the ventral route, whereas the dorsal route is involved primarily in the sensory-motor mapping of sound to articulation. These findings are consistent with a role for ventrolateral PFC auditory neurons in analyzing the features of auditory objects. Although the paired-click task is passive, an explanation of the present findings is that the ‘what’ stream (i.e., the auditory STG to IFG pathway) is activated in HC even when passively encoding auditory stimuli, with decreased activation of this pathway in SZ consistent with a deficit in encoding auditory stimuli (resulting in decreased STG auditory response), with such encoding deficits possibly leading to impairments in higher-order cognitive processes (Smith et al., 2010). In contrast, given abnormal activation in individuals with SZ in right medial SFG areas, individuals with SZ may abnormally activate the dorsal ‘where’ pathway (or perhaps language pathways involved in mapping phonological information with the frontal articulatory system).

Decreased STG and PFC gray matter in SZ (Gur et al., 2000; Mitelman and Buchsbaum, 2007; Olabi et al., 2011; Shenton et al., 2001; Thoma et al., 2004) may account for the decreased STG and PFC activity observed in the present SZ sample. Gray-matter reductions in SZ are thought to be due to elimination of the neuropil between neuron bodies (the reduced neuropil hypothesis) (Selemon and Goldman-Rakic, 1999). Sweet et al. (2003) found that, within auditory cortex, mean somal volumes of deep layer 3 pyramidal cells in BA 41 and 42 were reduced in SZ, and Sweet et al. (2007) observed reduced axon terminal densities in feed-forward auditory pathways. A combined MEG and proton magnetic resonance spectroscopy study showed that the number and integrity of neurons (assessed via auditory cortex N-acetylaspartate) and the density and functional integrity of cell membranes (assessed via auditory cortex choline-containing compounds) are associated with M100 source strength (Soros et al., 2006). Such abnormalities, perhaps leading to an abnormal spread of activity within cortical auditory areas after auditory stimulation, may explain the reduced STG and PFC activity observed in SZ.

In addition to STG and frontal group differences, the present study found group differences in left SMG (Figs. 3 and 4). SMG is located at the temporoparietal junction and links posterior auditory cortex with parietal regions via an auditory dorsal pathway (Hickok and Poeppel, 2000), a finding that again provides evidence that individuals with SZ abnormally engage fronto-temporal auditory dorsal pathways when processing auditory information. Previous studies have shown that enhanced SMG activity is associated with auditory hallucinations. Lewis-Hanna et al. (2011) found that healthy controls who experienced auditory hallucinations showed greater left SMG activity than controls not experiencing auditory hallucinations. Elevated left SMG activity has been observed during auditory hallucinations in SZ (Diederen et al., 2012; Sommer et al., 2008), with the severity of auditory hallucinations in SZ associated with volume loss in left SMG as well as in left Heschl's gyrus and right IFG (Gaser et al., 2004). In the present study, post hoc analyses showed no associations between SMG activity and PANSS measures of auditory hallucinations. As the individuals with SZ recruited for this study were stable and mostly likely not hallucinating at the time of the MEG scan, this may explain the failure to observe SMG and PANSS associations.

Finally, and unexpectedly, posterior SFG/SMA activity was observed in both groups. A review of the literature indicates, however, that SMA areas are part of the auditory–motor integration network, important for speech production (Hickok and Poeppel, 2000). Although previous studies suggest involvement of SMA during auditory tasks, replication of this finding is needed.

A limitation of the present study is that most subjects were chronic patients and all patients were on medication. As such, it is not possible to determine whether the observed abnormalities are observed only in chronic patients or whether group differences were due to medication. Several studies, however, show that 100 ms abnormalities in schizophrenia are present at first onset as well as in first-degree relatives (Turetsky et al., 2008), suggesting that auditory abnormalities in SZ are not due to medication. In addition, in a review of 100 ms auditory studies Rosburg et al. (2008) concluded that medication did not seem to account for group differences in N100 activity.

In sum, present findings indicate that early auditory encoding abnormalities in SZ are not limited to STG and that there are abnormalities in multiple nodes of a concurrently activated auditory network. Present findings suggest that individuals with SZ show decreased activation in a temporal to frontal ventral pathway (a possible auditory ‘what’ pathway) as well as abnormally increased activation in a temporal to frontal dorsal pathway (a possible auditory ‘where’ pathway). As detailed earlier in the Discussion, although the paired-click task is passive, an explanation of the present findings is that the ‘what’ stream (the auditory STG to IFG pathway) is activated in HC even when passively encoding auditory stimuli, with decreased activation of this pathway in SZ consistent with a deficit in encoding auditory stimuli (resulting in decreased STG auditory response).

The following are the supplementary data related to this article.

**Supplementary Fig. 1:**
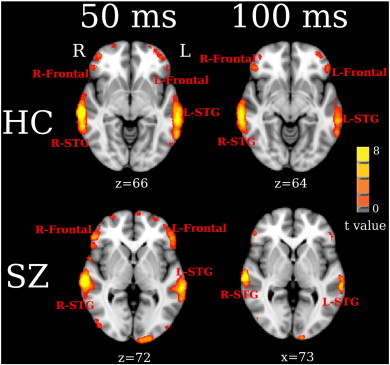
Within-group VESTAL statistics for 50 (left panel) and 100 ms (right panel) S2 activity showing left and right STG (L-STG, R-STG) and frontal regions (L-Frontal, R-Frontal) activated areas for HC (top panel) and SZ (bottom panel) (thresholded at FDR q < 0.01).

**Supplementary Fig. 2:**
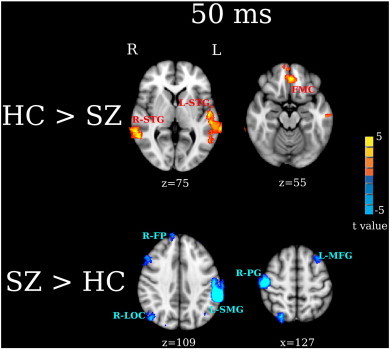
Between-group analyses for 50 ms S2 activity. Activation clusters in yellow/red (thresholded at FDR q < 0.05) show stronger L-STG, R-STG, and frontal medial cortex (FMC) activity in HC than SZ (HC > SZ). Activation clusters (thresholded at FDR q < 0.05) in blue show stronger activity in left middle frontal gyrus (L-MFG), L-SMG, right lateral occipital cortex (R-LOC), right frontal pole (R-FP), and right postcentral gyrus (R-PG) in SZ than HC (SZ > HC).

**Supplementary Fig. 3:**
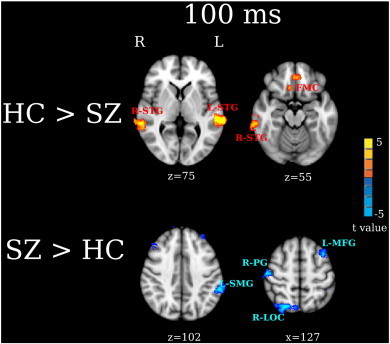
Between-group analyses for 100 ms S2 activity. Activation clusters in yellow/red show stronger activity in L-STG, R-STG, and FMC in HC than SZ (HC > SZ). Activation clusters in blue show stronger activity in L-SMG, L-MFG, R-LOC, R-PG in SZ than HC (SZ > HC).

Supplementary data to this article can be found online at http://dx.doi.org/10.1016/j.nicl.2013.05.002.

## Figures and Tables

**Table 1:** Demographic information of HC and individuals with SZ.

	HC (N = 22)		SZ (N = 19)	
	Mean	SD	Mean	SD
Age	34.95	10.2	40.31	11.7
Education (years)	13.7	1.16	13.5	2.15
SES ⁎	57.4	12.68	64.83	7.51
Parental SES	44.45	18.46	44.64	19.92

⁎ HC had higher SES, t(36) = − 2.22, *p* < 0.05. Group differences in age, t(39) = − 1.57, education t(39) = 0.36, and parental SES, t(36) = − 0.03, were not significant (*p*s > 0.12).

**Table 2:** Effect size (Cohen's d) for L-STG, R-Frontal, and R-SFG.

MEG VESTAL measures	Cohen's d
L-STG M50	1.19
L-STG M100	1.29
R-Frontal M50	1.13
R-Frontal M100	1.16
R-SFG M50	− 0.57
R-SFG M100	− 0.71

## Appendix A

In VESTAL (Huang et al., 2006), we take the lead-field based MEG source imaging approach and divide the source space (the brain volume or just the cortex) into a grid of a large number of dipole locations. The *M* × *N* sensor waveform matrix *B*(*t*) = [*b*(*t*_1_), *b*(*t*_2_), …, *b*(*t*_*N*_)] contains MEG data where *m* is the number of MEG sensors and *s* is the number of time points, *b*(*t*_*i*_) is an *M* × 1 vector of the MEG measurements at given time point. For each column of *B*, we have:

(1) $$B\left(t\right)=\mathrm{GQ}\left(t\right)+\mathrm{Noise}\left(t\right)$$

where **G** is an *M* × 2*P* gain (lead-field) matrix calculated from MEG forward modeling for the pre-defined source grid with *P* dipole locations, with each dipole location having two orthogonal orientations (i.e., θ and ϕ). **Q**(*t*) = [*q*(*t*_1_), *q*(*t*_2_), …, *q*(*t*_*N*_)] is a 2*P* × *N* source time-course matrix. In the spherical MEG forward head model, θ and ϕ represent the two tangential orientations for each dipole location. In the present study, a realistic MEG forward model using the boundary element method (BEM) is used to calculate **G** (Huang et al., 2007; Mosher et al., 1999), and in BEM the θ and ϕ-orientations are obtained as the two dominant orientations from the singular-value decomposition (SVD) of the *M* × 3 lead-field matrix for each dipole, as previously documented (Huang et al., 2006). The BEM mesh was obtained from tessellating the inner skull surface from the MRI into ~ 6000 triangular elements with ~ 5 mm size. The noise term in Eq. (1) is assumed to be Gaussian white noise. If correlated noise exists, pre-whitening procedures can be applied (Huang et al., 2006; Mosher et al., 1999; Sekihara et al., 1997). The inverse solution in Eq. (1) obtains the source time-courses **Q**(*t*) for given MEG sensor wave-forms *B*(*t*). In general, for each time-sample, since the number of unknown parameters is far greater than the number of sensor measurements (i.e. 2*P* ≫ *M*), MEG source imaging is dealing with a highly under-determined problem, and there are a large number of solutions that fit the data. To reduce the ambiguity, additional constraints (source models) are needed.

The conventional minimum L1-norm solution selects the source configuration that minimizes the absolute value of the source strength. Let **G** = **USV***^T^* be the singular value decomposition of the gain matrix, the minimum L1-norm *q* that meets the following condition (Sekihara et al., 1999):

(2) $$\min\left(w^{T}\left|q\right|\right),\;\mathrm{subject}\;\mathrm{to}\;\mathrm{constraints}\;S_{\mathrm{ng}}V_{\mathrm{ng}}^{T}q≃U_{\mathrm{ng}}^{T}b$$

where *S*_*ng*_, *U*_*ng*_, and *V*_*ng*_ contain the *ng* largest singular values and the associated singular vectors, respectively. In Eq. (2), *w* is an 2*P* × 1 optional weighting vector chosen to remove potential bias towards grid nodes at the superficial layer and it is usually taken to be the column norm of the **G** matrix (Matsuura and Okabe, 1997; Uutela et al., 1999) or a Gaussian function (Ioannides et al., 1993). A major limitation with previous L1-minimum norm routines has been instability in spatial localization and poor smoothness in reconstructed source time-courses. The time-course of one specific grid point can show substantial spiky-looking discontinuities. This problem is also encountered in other focal localization methods using lead-field approaches.

To increase spatial and temporal stability, we developed a spatial–temporal vector-based minimum L1-norm solution (i.e., VESTAL). The idea was based on a principle of MEG physics, which states that the magnetic waveforms in the sensor-space are linear functions of the dipole time-courses in the source-space. If we perform singular value decomposition for the *M* × *N* MEG sensor waveform data matrix:

(3) $$B=U_{B}S_{B}V_{B}^{T}$$

one can see that all temporal information in the MEG sensor waveform can be represented as a linear combination of the singular vectors in the matrix *V_B_*. Since MEG sensor waveforms are linear functions of the underlying neuronal source time-courses, the same signal sub-space that expands the temporal dimension of *B* should also expand the temporal dimension of the 2*P* × *N* source time-course matrix **Q** = [*q*(*t*_1_), *q*(*t*_2_), …, *q*(*t*_*N*_)] estimated from the vector-based minimum L1-norm solution for *s* time points. In the present study, **Q** was solved using SeDuMi package (http://sedumi.ie.lehigh.edu/). By projecting **Q** towards *V_B_* we can ensure that source time-courses matrix **Q** and sensor waveform matrix *B* share the same temporal information as requested by the MEG physics:

(3) $$Q_{\mathrm{VESTAL}}=QP_{\left|\right|}$$

where the projection matrix **P**_||_ = *V*_*B*_*V*_*B*_^*T*^ is constructed using the dominant (signal-related) temporal singular vectors (subspace) of the sensor waveforms. We called **Q***_VESTAL_* the Vector-based Spatial-temporal Analysis using L1-minimum-norm (VESTAL). In VESTAL, the temporal information in the data is used to enhance the stability of the reconstructed solution. Since this approach makes no assumptions about the temporal dynamics of the sources, the approach can handle sources that are 100% correlated. More technical details of VESTAL, in which VESTAL was tested with computer simulations and human data, are presented in Huang et al. (2006). Results show that VESTAL provides high spatial stability and continuous temporal dynamics, without compromising spatial or temporal resolution.

## Footnotes

1 Within-group and between-group VESTAL group statistics for S2 are provided in Supplementary Figs. 1–3.

2 Although many paired-click studies have examined the association between frontal activity and gating measures (e.g., second click divided by first click), studies examining associations between paired-click ratio scores and frontal activity but not also associations between first click activity and frontal activity are not discussed as findings between ratio scores and frontal activity are outside the scope of this study (e.g., EEG studies such as Williams et al. (2011) and fMRI studies such as Tregellas et al. (2007) and Mayer et al. (2009)).
